# Analytical strategies for clinical studies on dental erosive wear

**DOI:** 10.1186/s12903-019-0834-1

**Published:** 2019-07-26

**Authors:** K. R. Stenhagen, B. Holme, A. B. Tveit, A. Lussi, T. S. Carvalho

**Affiliations:** 1 Department of Cariology and Gerodontology, Faculty of Dentistry, University of Oslo, Blindern, NO-0316, Postbox 1072 OSLO, Norway; 2 The Public Dental Service Competence Centre of Northern Norway, TkNN, Tromsø, Norway; 3 SINTEF Industry, Forskningsveien 1, 0373 Oslo, Norway; 4 Asker, Norway; 5 Department of Restorative, Preventive and Pediatric Dentistry, University of Bern, Freiburgstrasse 7, CH-3010 Bern, Switzerland

**Keywords:** Erosive tooth wear, Clinical studies, Measurements

## Abstract

**Background:**

There is a need for analytical techniques for measuring Erosive Tooth Wear (ETW) on natural surfaces in clinical studies. The purpose was to investigate the use of two instruments aimed to assess initial to more advanced stages of ETW.

**Methods:**

Human premolar enamel samples (2x3mm) (*n* = 24), were polished flat and mounted in resin cylinders (4 cylinders, 6 samples in each). Part 1: Baseline analyses by White Light Interferometer (WLI), Surface Reflection Intensity (SRI: TableTop and OptiPen) and Surface Hardness (SH). Erosion (1% citric acid (pH 3.6) for 1, 2, 4, 6, 8, 10 min. SRI and SH analyses after every erosion episode and by WLI after 10 min. New indentations were made and enamel loss; was measured by change in indentation depths from toothbrush abrasion (200 g, 60 strokes, 30 s). Another series of 2 × 5 min erosion (totally15 min and 20 min) was analysed with SH and SRI after each erosion, and by WLI (on samples and impressions of samples) after 20 min. Part 2 investigated WLI performance in the interface where initial erosion increases in severity and substance loss occurs. The samples were repolished. Baseline analyses by WLI, SRI (TableTop and OptiPen) and SH. Four cylinders were etched for 1, 2, 4, 8 min respectively and analysed by SRI, SH on samples, and WLI on samples and impressions).

**Results:**

Part1: SRI decreased from baseline to ~ 6 min etch and increased slightly after abrasion, the two devices correlated well (ICC 0.98 *p* < 0.001, Spearmans rs 0.91 *p* < 0.001). SH decreased nearly linearly to 10 min etch, but increased distinctly after abrasion. Mean enamel loss from abrasion alone was 0.2 μm (change in indentation depths). After 10 min etch, it was 0.27 μm (WLI) and after 20 min etch, it was 2.2 μm measured on samples vs 2.4 μm on impressions of samples (7% higher). Part 2: From baseline to 8 min etch; SRI and SH decreased whereas WLI presented increasing etch depths.

**Conclusions:**

With some adjustments, the use of SRI and WLI in combination seems to be a promising strategy for monitoring ETW in clinical studies.

## Background

The condition *erosive tooth wear* among young individuals is of growing concern. The erosive wear process is complicated, and modified by chemical, behavioral and biological factors in the oral cavity [1]. Longitudinal studies on incidence and progression of erosive lesions in individuals normally use erosion indexes to assess the severity of the lesions. However, such indexes do not detect changes on micrometer level. It may take years to detect changes in the severity of the lesions, from lesions on enamel to lesions reaching into dentin. So far, studies on interventions to control and inhibit tissue loss from erosive wear have been limited to in vitro and in situ studies. The reasons have been lack of methods suitable for assessing loss of dental hard tissue directly on the tooth surfaces in the mouth. Most analytical techniques have drawbacks; they require that tooth surfaces are ground flat to obtain good validity of the measurements or the technique is destructive and do not allow longitudinal repeated analyses [2]. One approach to solve these challenges has been the use of indirect techniques by measuring tooth wear on impressions or casts [3–8]. This requires a reference surface on the teeth, not affected by erosive wear. In addition, a technique that can give accurate measurements on slightly curved surfaces, like those on natural teeth, is required. Analyses of erosive enamel loss by a White Light Interferometer was previously validated, and shown to be accurate and precise, when measuring both flat and naturally curved surfaces [5, 9, 10]. It also gives reliable measurements on impressions of enamel surfaces [7], so this technique may be suitable for use in clinical studies (in vivo). The detection limit for reliable measurements by the WLI technique on natural enamel surfaces is~ 0.3 μm [11]. When analyzing enamel loss on impressions, the measurements depend on the accuracy of the impression material. It has been suggested that for in vivo trials using WLI and a two- component polymer impression material with less than 10% error for step heights above 1 μm, enamel loss ≥10 μm can be accurately measured [12]. This implies that this technique is relevant for advanced erosive lesions, and not the initial ones with loss of hardness due to superficial mineral loss only.

Quite recently, a technique has been developed that measures changes in reflection intensity on enamel surfaces by the use of an optical device; reflectometer (SRI). It has been validated and showed promising results for measurements on both, ground and natural surfaces [13–16]. Also a smaller version of the instrument; a pen-size device has been developed that can be used directly on the natural tooth surfaces [17] in the oral cavity. In order to investigate the clinical performance of the device (SRI), a validation against registration of dental erosive wear by the Basic Erosive Wear Examination (BEWE) in vitro on extracted teeth has been performed [18]. The reflectometer correctly diagnosed erosive tooth wear (sensitivity ≥64%) on the permanent teeth, somewhat better for advanced lesions (BEWE ≥2) than for milder cases (BEWE 1). The specificity was high (≥ 84%). More importantly, the authors emphasized that the reflectometer does not measure the amount of hard tissue already lost by erosive tooth wear, so it will not substitute clinical diagnosis.

It is important to develop methods that could quantify the actual mineral loss in the mouth due to the erosive wear process [1]. SRI is reliable for identifying erosive tooth wear, but cannot give information about the amount of enamel that is lost. WLI can analyse enamel loss both on natural surfaces and on impression of these surfaces. Considering the strengths and limitations of the WLI and SRI techniques, we hypothesize that they could supplement each other in clinical studies.

The aim was therefore to investigate the capability of SRI and WLI to monitor the surface loss from initial to more advanced stages of erosive tooth wear. How the instruments performed in the transition phase where initial erosion with mineral loss in the surface layer increases in severity and substance loss occurs was of particular interest.

## Methods

### Enamel specimens

Twelve extracted caries-free human premolars were collected and kept in individual containers with thymol crystals and cotton rolls soaked in water in a Biobank at the Faculty of Dentistry, University of Oslo, Norway until use (Approved by the Norwegian Institute of Public Health’s Biobank Register No. 2543, project No. 6.2008.2058). Two circular cavities filled with amalgam (~ 1 mm in diameter) were made ~ 1.5 mm from the enamel-root junction. The amalgam filling serves as reference surface during the analyses of enamel loss by the White Light Interferometer (WLI) since it has proven to be undisturbed by acid etching [5]. Two enamel specimens (~2x3mm), including the amalgam reference surfaces, were made from each tooth (*n* = 24). All specimens were embedded into resin cylinders (Paladur, Heraeus Kulzer GmbH, Hanau, Germany); 4 cylinders with 6 specimens each custom-made to fit into all analytical instruments.

The surfaces were serially ground and polished under constant tap-water cooling using a Knuth Rotor machine (LabPol 21, Struers, Copenhagen, Denmark) with silicon carbide paper discs of grain size 18.3 μm, 8 μm, 5 μm, as well as using a 3 μm diamond abrasive. Each paper disc was used for 60 s (LaboPol-6, DPMol Polishing, DP-Stick HQ, Struers, Copenhagen, Denmark). Between the grinding steps and after the final polishing, all specimens were sonicated for 1 min in tap water and rinsed. All prepared specimens had a flat ground enamel area with a 200 μm cutoff layer. The samples were stored in a mineral solution (1.5 mmol/L CaCl_2_, 1.0 mmol/L KH_2_PO_4_, 50 mmol/L NaCl, pH 7.0) [19]. Immediately before the experiment, the samples underwent further polishing with a 1 μm diamond abrasive (60 s, LaboPol-6, DP-Mol Polishing, DP-Stick HQ, Struers, Copenhagen, Denmark).

### Analytical techniques

#### Surface relative reflection (SRI) (table model and hand held OptiPen)

Surface Reflection Intensity of the enamel surfaces was measured using two devices: one hand-held pen-size reflectometer (OptiPen) and one Table-Top reflectometer. Both devices were connected to a computer, running a specific software program used to register SRI. Initially, we measured SRI in all samples using the Table-Top reflectometer. For those measurements, the blocks were individually placed on a platform (sample holder) under a laser beam, the source of which is fixed to the reflectometer. As previously described [16], the laser beam is adjusted on the sample surface by moving the holder in the *z*-axis (height). The point of highest reflection intensity is then registered, which represents the best positioning of the sample in relation to the device. Later, we measured SRI using the OptiPen reflectometer. This pen-sized device functions in a similar manner, but the laser beam derives from the hand-held piece, the tip of which is placed directly onto the enamel surface. In the same way as the Table-Top device, the laser from the hand-held reflectometer is also adjusted on the surface of the sample by slightly inclining the device in different angles. The point of highest reflection intensity is then registered [17]. For both the Table-Top and OptiPen reflectometers, the point of highest reflection intensity is expressed as an SRI value, which represents the best positioning of the sample in relation to the reflectometer. Highest SRI value corresponds to non-eroded polished enamel surface, and as the enamel surface is eroded, the SRI value decreases.

The reflectometers are each fitted with a laser diode (oeMarket, Cherrybrook, Australia), which emits a laser beam (635 nm) onto the surface of the sample. The reflected light is then captured and measured with a photodiode (FDS100, Thorlabs, Dachau, Germany). The Table-Top device has a beam incidence angle of 45°, whereas the OptiPen device has an angle of incidence if ~ 23°.

For the statistical analyses, we calculated the relative SRI (*rSRI*) for each reflectometer. Since SRI measurements were carried out using both reflectometers at baseline (*SRI*_*0*_) and after each erosive challenge (*SRI*_*t*_), we calculated the *rSRI* for both devices using the formula: *rSRI* = (*SRI*_*t*_ / *SRI*_*0*_) × 100%.

#### Surface hardness (SH)

The initial surface hardness measurements (*SH*_*0*_) were calculated from six indentations made at intervals of 50 μm. After each erosive challenge, six further indentations were made next to the previous ones, and the new surface hardness value was calculated (*SH*_*t*_). We used nanoindentations made with a Vickers diamond under a pressure of 50 mN for 15 s (Fischerscope HM 2000 XYp; Helmut Fischer, Hünenberg, Switzerland) and Vickers hardness values were automatically calculated from the depth of the indentations by the computer program. The device allowed fully automatic measurements using a programmable *x-y* stage, and software WIN-HCU. The *SH*_*0*_ and *SH*_*t*_ values for each enamel slab were determined by calculating the average of the six indentations at baseline and after erosive challenge, respectively. For statistical analyses, we calculated the *rSH* for both devices using the formula: *rSH* = (*SH*_*t*_ / *SH*_*0*_) × 100%.

#### White light interferometer (WLI)

All specimens were imaged at baseline by a White Light Interferometer (WYKO NT9800; Veeco, Tucson, Ariz., USA), which is a computerized optical interference microscope operating in the vertical scanning interferometry mode, suitable for measuring enamel loss on flat and naturally curved tooth surfaces [5]. Subtraction of the baseline images from the images taken after 10 min etch and after 20 min etch for every separate specimen, created the respective difference images showing the enamel loss in the sampling area (∼1 mm^2^). The objective had a × 10 magnification and a field-of-view selector of 0.55, giving a pixel size of 3.6 μm. The image size was 1.1 mm by 0.85 mm, with a pixel resolution of 320 by 240 pixels. A validation of the technique reported a 0.1 μm precision, 0.05 μm accuracy and 0.3 μm detection limit for step height measurements on etched, naturally curved enamel surfaces [5, 9–11].

By making a WLI image of the impression of the specimen surface and inverting the data, a 3D image similar to that of the specimen was created. The inverted impression images were analysed in the same way as the images of the enamel surfaces in order to measure the step height in the difference images.

### Experimental procedures

**Fig. 1:**
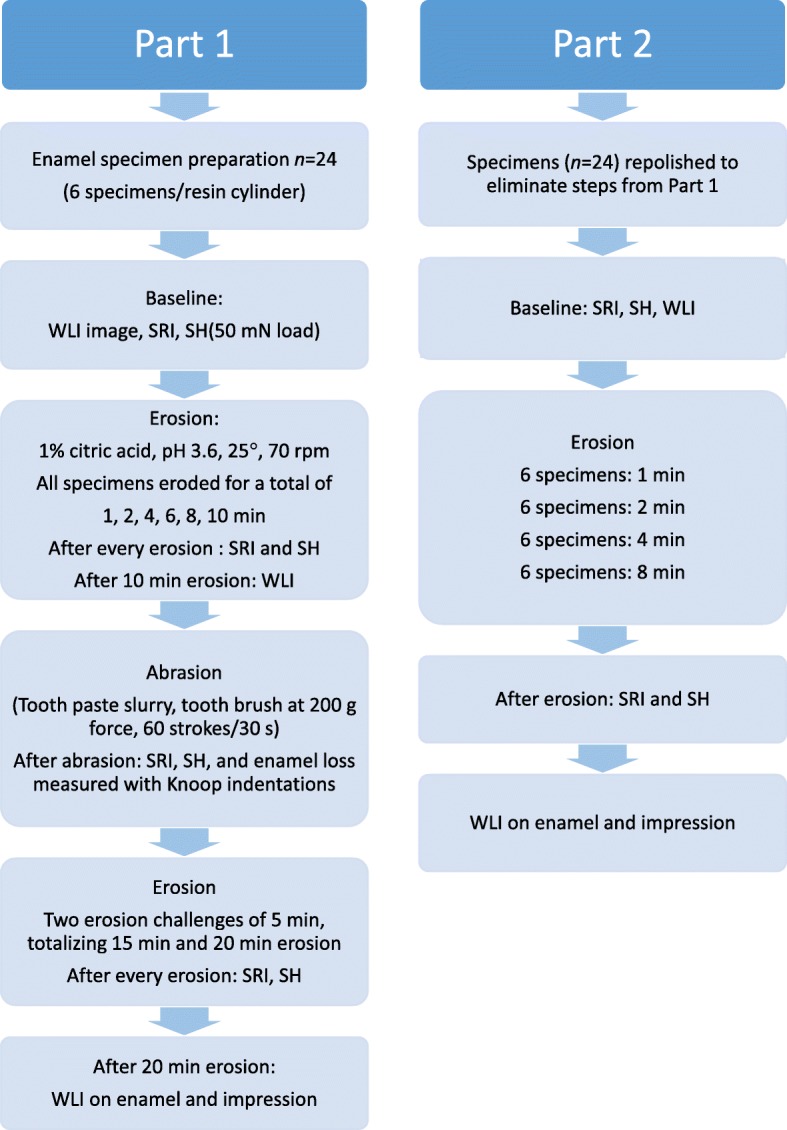
Procedures for Part 1 and Part 2. Surface Reflection Intensity (SRI) was always measured by both the TableTop and OptiPen device. Enamel loss from toothbrush abrasion (Part 1) was calculated from changes in indentation depths from the hardness instrument. Enamel loss from erosion was measured by a White Light Interferometer (WLI). In Part 2, SRI, SH and WLI analyses were made after 1, 2, 4, 8 min etch on the respective cylinders

A flow chart presents the procedures for Parts 1 and 2 in Fig. 1.

### Part 1

At baseline, surface hardness values (SH), surface reflection intensity (SRI; with TableTop and OptiPen) and surface topography images (WLI) were obtained.

The specimens were etched using 50 mL of 1% Citric Acid (≥ 99.5%, Merck KGaA, Darmstadt, Germany). A series of erosive challenges for a total of; 1, 2, 4, 6, 8 and 10 min (pH 3.6, 25 °C, shaking 70 rpm with travel-path 22 mm) were carried out. Analyses of SH and SRI (TableTop and OptiPen) were then performed after 1, 2, 4, 6, 8 and 10 min erosion respectively, but WLI analyses not until after 10 min etch (before abrasion).

In order to evaluate enamel loss from abrasion only, the specimens were then brushed for 30 s; 200 g load and 60 strokes with toothbrush and toothpaste slurry. Changes in reflection and hardness after abrasion was measured by SRI (TableTop and OptiPen) and by SH. Enamel loss from toothbrushing was measured using the indentation method [20], where six Knoop indentations were made on the enamel surface before the abrasive challenge and their lengths were measured before and after the abrasion. From the difference in length, we were able to calculate the difference in indentation depth.

Another series of 2 × 5 min erosive challenges totalized the etching time for 15 min and 20 min respectively with analyses of SH and SRI after each etching respectively. Enamel loss was measured after 20 min etch both on the enamel specimens and on impressions of the surfaces by WLI. The impression material was a two-component polymer with the ability to reproduce features down to 0.1 μm (Microset® 101TH, Microset Products Ltd., Warwickshire, UK; [21]) and applied by a mixing pistol, similar to the type used to apply dental impression materials.

### Part 2

The purpose of this follow-up study, performed on the same samples, was to investigate in more detail how WLI performs and gives sensitive quantification of the erosive process in the transition phase where initial erosion with mineral loss in the surface layer increases in severity and substance loss occurs. This is the phase where the SRI works best but falls rapidly as the erosive process progresses.

All specimens (6 specimens/cylinder in 4 cylinders) were polished (same procedure as in Part 1) in order to eliminate the etch step formed during Part 1. The specimen surfaces were evaluated by a microscope to assure the step was removed by the re-polishing and no dentin was exposed.

At baseline, SRI and SH analyses and WLI images were made on all 24 specimens. The specimens were etched using 50 mL of 1% Citric Acid (≥ 99.5%, Merck KGaA, Darmstadt, Germany). as follows; 6 specimens were etched for 1 min, 6 specimens for 2 min, 6 specimens for 4 min and 6 specimens for 8 min. SRI, SH, and WLI analyses were performed after these respective erosion durations on all specimens, and WLI analyses were also performed on impressions of the specimen surfaces.

### Statistical analyses and validation of instruments

Previous validation studies have shown that relative SRI loss from TableTop and OptiPen are similar, and significantly correlated to relative SH loss (*r*_*s*_ = 0.8 *p* < 0.001) [14]. It has been shown that measurements of enamel loss by WLI on enamel and impression surfaces correlate well (*r*^*2*^ = 0.95, *p* < 0.05) with 7% higher values on impressions [7].

The agreement between relative SRI TableTop and OptiPen values from Part 1, is expressed by Intra-class Correlation Coefficient ICC, using a two- way random model with measures of consistency and by Spearmans correlation coefficient values (*r*_*s*_).

## Results

### Part 1

#### SRI and SH

**Fig. 2:**
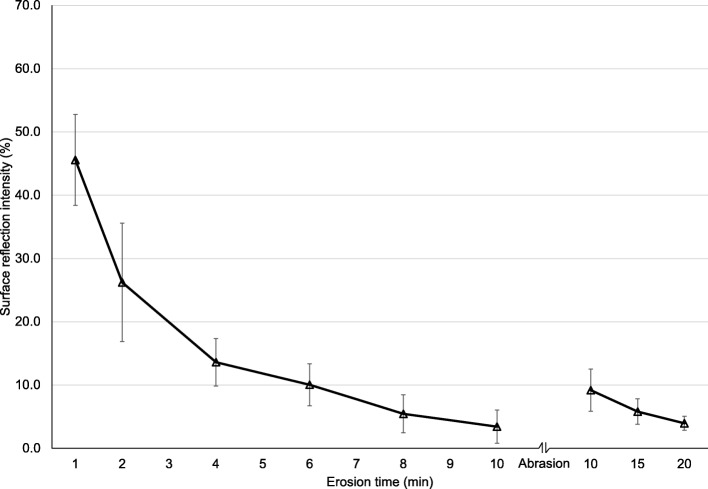
Part 1. Percentage surface reflection intensity (SRI) by the Tabletop Device after 1, 2, 4, 6, 8 and 10 min etch of the enamel specimens, after abrasion and after 15 min and 20 min etch. The error bars represent 1 standard deviation

**Fig. 3:**
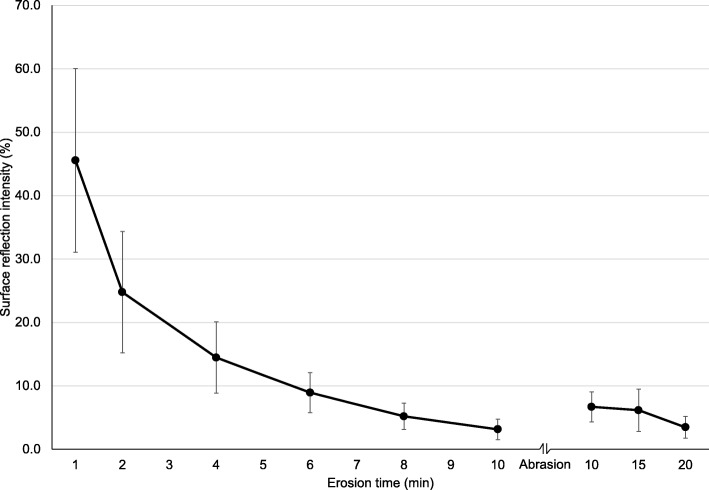
Part 1. Percentage surface reflection intensity (SRI) by the OptiPen after 1, 2, 4, 6, 8 and 10 min etch of the enamel specimens, after abrasion and after 15 min and 20 min etch. The error bars represent 1 standard deviation

Average (SD) surface reflection intensity measured by SRI TableTop and OptiPen agreed very well ICC = 0.98, *p* < 0.001, Spearmans *r*_*s*_ = 0.91, *p* < 0.001. Baseline reflection values were 34.6 (1.9) vs 36.1 (5.1), for TableTop and OptiPen, repectively. The SRI values dropped after 1 min etch from 100 to 45.6% (±7.2%) and 45.6% (±14.5%), for TableTop and OptiPen, respectivley. The values decreased further after every erosive challenge, and after 10 min they had dropped to 3.4% (±2.6%) and 3.1% (±1.6%). After the abrasion, SRI increased to 9.2% (±3.3%) and 6.7% (±2.4%), and again decreased, ending at 4.0% (±1.1%) and 3.5% (±1.7%) after 20 min erosion, for TableTop and OptiPen, respectively **(**Figs. 2 and 3).

**Fig. 4:**
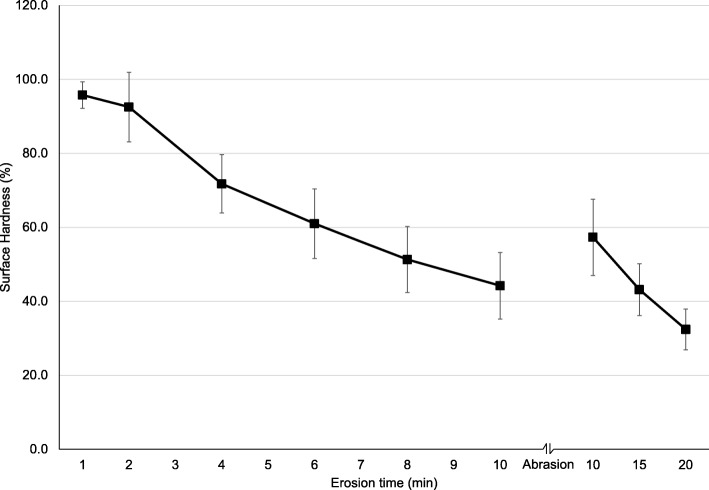
Part 1. Percentage surface hardness values (SH) after 1, 2, 4, 6, 8 and 10 min etch of the enamel specimens, after abrasion and after 15 min and 20 min etch. The error bars represent 1 standard deviation

Mean (SD) SH at baseline was 516 (24) Vickers Hardness Number (VHN). SH decreased almost linearly from 100 to 44.2% (±9.0%) after 10 min etch **(**Fig.4). After the toothbrush abrasion, mean SH (SD) increased to 57.3% (±10.3%), and further decreased to 32.4% (±5.5%) after 20 min etch.

#### Enamel loss from toothbrush abrasion measured using the indentation method

Mean enamel loss per tooth from toothbrush abrasion was 0.20 μm (range 0.01–0.31 μm measured from the difference in indentation depths at baseline 1.83 μm (0.07 μm) and after abrasion 1.66 μm (0.06 μm), as described in [20].

#### Enamel loss measured by WLI on enamel and on impressions

Mean (SD) enamel loss measured by WLI after 10 min etch was 0.3 μm (0.3 μm). After 20 min etch it was 2.2 μm (1.4 μm) measured on enamel surfaces and 2.3 μm (1.4 μm) measured on impressions of the same surfaces, that is 7.3% higher on the impressions. The somewhat large standard deviations reflect significant differences in the etch depth measured on the different samples.

### Part 2

**Fig. 5:**
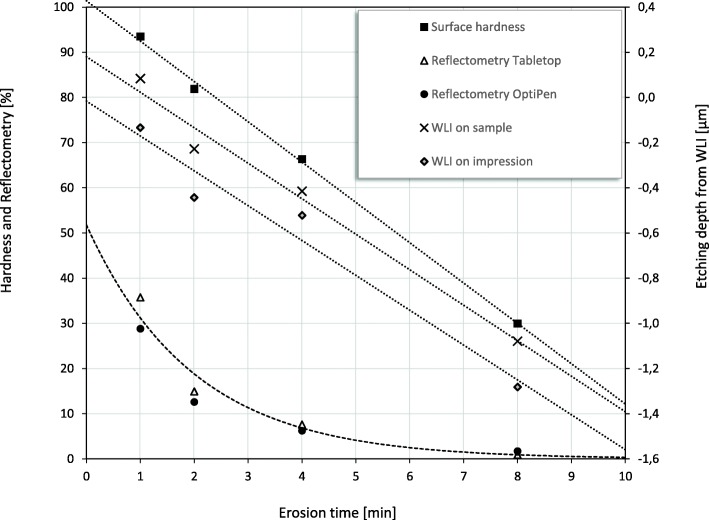
Part 2. Percentage measurement values for cylinder 1 (1 min etch), cylinder 2 (2 min etch), cylinder 3 (4 min etch) and cylinder 4 (8 min etch); by SRI (TableTop and OptiPen), SH (% scale on the y-axis to the left). Average enamel loss (μm) by WLI on enamel sample and WLI on impression are shown (downward scale from zero to increasing enamel loss expressed by negative eth depths) on the y-axis to the right). The dotted and dashed lines represent the trend lines for the registrations by SH, SRI and WLI, respectively

Figure 5 presents all results from Part 2. Enamel loss measured by WLI increased nearly linearly from zero to 1.1 μm during 1–8 min etch (y-axis to the right in Fig. 5 where negative values represent enamel loss). These changes on the enamel surfaces represent initial erosion where the surface is demineralized and softer, as shown by the correspondingly decreasing SH values that went from 94% after 1 min etch to 30% after 8 min etch. The reflection intensity (both devices averaged) decreased from 1 min to ~ 4 min etch (corresponding to 0.4 μm etch depth by WLI) from 32 to 7% reflectivity. When etch depths reached ~ 1 μm (after ~ 8 min etch), the reflectivity was very low and levelled out. This indicates that a quite rough, steady-state surface topography had been reached. Etch depths by WLI on impressions of the enamel specimen surfaces shows typically higher etch depths (average difference 0.2 μm) than when measured on enamel.

## Discussion

The established fact that erosive tooth wear is highly prevalent, ~ 30% in children and adolescents, has led to extensive research into risk assessment and causal treatment, but also into therapeutic agents. The importance of development of new diagnostic technologies to overcome the challenges of research in clinical settings has been emphasized [1].

When looking at the incentive and need for clinical studies of erosive tooth wear, several approaches are relevant. First, it is important to develop effective prevention methods to avoid structural enamel loss, also concluded in a recent study of 3D surface texture characterization [22]. This implies the need for instruments that are sensitive to the initial stages of erosion on natural surfaces in vivo, like SRI OptiPen. Secondly, there is a need for longitudinal clinical studies to monitor increments and progression of erosive lesions in young patients at risk or in particular risk groups. Thirdly, there is a need for longer observation periods to monitor if erosive lesions progress related to onset of causal preventive measures and/or to intervention with preventive vehicles.

Taking these challenges in clinical studies into consideration, the main purpose of the present study was to investigate the performance of SRI and WLI from initial to more advanced stages of erosive tooth wear (Part 1). How the instruments performed in the transition phase where initial erosion with mineral loss in the surface layer increases in severity and substance loss occurs was of particular interest (Part 2).

The results from Part 1 and Part 2 indicate that measurements of surface reflection gives information about initial stages of enamel erosion where the surface has softened due to acid induced mineral loss. This is a result of less reflection due to the etch pattern on the surface after an acidic challenge, which gives increased roughness [14]. Decreasing surface hardness values from baseline during the present protocol confirmed a softening of the surface layer (Fig. 4). Studies have reported that the loss of dental hard tissue during the initial stage of erosion ranges from a few nanometers to a few micrometers [20, 23, 24]. When the erosion process reaches advanced stages where the enamel crystals dissolve and surface loss occurs, the reflection measurements (SRI) are less sensitive [14]. During the present protocol, that stage occurred for both SRI instruments after ~ six minutes erosion by citric acid (Figs. 2 and 3). The protocol for Part 1 did not include enamel loss measurements by WLI after 6 min etch, but after 10 min where the enamel loss was 0.3 μm.

In previous studies where the WLI technique was developed and validated for analyzing enamel erosive wear, a different erosion protocol was used [5, 9–11]. In those studies, the erosion fluid was hydrochloric acid in order to mimic episodes where the gastric acid comes into the oral cavity. The etch rate was consistently on average ~ 1 μm per minute erosion. In contrast, the citric acid used in the present study imitates intake of acidic drinks/foods and is less aggressive since it has a considerably lower etch rate in the advanced stages; 0.1 μm enamel loss per minute erosion calculated from WLI values after 20 min of erosion. Therefore it was interesting to investigate how the WLI technique could detect such small etch depths, since the detection limit previously has been validated to be ~ 0.3 μm [11]. The quantification of enamel loss by WLI after 1–2 min etch in part 2, may represent some uncertainties since the values are in the area of the detection limit of WLI on etched enamel. However, it was relevant to get an indication of the enamel loss even at such initial stage in the erosion process. In Fig. 5 where the results for both SRI (both devices), SH and WLI (Part 2) are presented, the etch depths measured on enamel (WLI) increased nearly linearly up to 1.1 μm from 1 min to 8 min etch. The respective measurements (WLI) on impressions presented typically 7% higher etch depths (average difference 0.2 μm) after 20 min erosion, consistent with previous validations [7]. In that study, the conclusion was that taking measurements from the impressions is relevant for advanced erosive lesions and not the initial ones. The reason is that the accuracy of the impression material may be the limiting factor. Preliminary studies suggested that for in vivo trials using WLI and a two-component polymer impression material with less than 10% error for step heights above 1 μm, enamel loss ≥10 μm can be accurately measured [12]. The impression material used in the present study has higher accuracy than most conventional dental impression materials. It is able to detect features down to 0.1 μm and is suitable for use in vivo. In addition, there is a need for a proper reference point for the impression technique, represented by the amalgam filling in the present study. In an overview of techniques for erosion studies, a few instruments were useful for native surfaces in vivo [2]. One such technique was Optical Coherence Tomography (OCT) which is non-destructive, but it is expensive and only suitable for early erosive lesions. The OCT was able to detect and quantify progression of dental tissue demineralization after treatment with a proton-pump inhibitor in patients with gastroesophageal reflux disease (GERD) [25], but the authors pointed out the need for further studies.

In clinical settings, teeth are exposed to tooth brushing, abrasion from the tongue and hard food items, leading to modification of the softened layer and increased SRI values due to a smoother “polished” surface vs healthy enamel with intact perikymata that appear rougher in comparison [14]. This was confirmed in the present study where mean surface hardness increased after the toothbrush abrasion (Fig. 3). This implies that abrasion removed the demineralized softened surface layer, thus presenting the underlying harder layer. In fact, toothbrush abrasion removed 0.2 μm measured by change in nano-indentation depths, confirmed by increased reflectivity (higher SRI values Figs. 2 and 3).

When the condition is clinically visible, the use of a validated severity index is relevant, like the Basic Erosive Wear Examination system (BEWE) [26, 27] or Visual Erosion Dental Examination system (VEDE) [28]. A significant association was found between BEWE and SRI [18] and it was concluded that SRI was able to distinguish eroded native enamel surfaces on permanent teeth. However, it gives no information about the severity and degree of enamel loss. This implies the need for a supplemental technique able to measure substance loss on eroded natural tooth surfaces.

Another important factor to take into consideration is that the use of SRI, has not been tested in patients with severe erosive tooth wear with dentin exposed. On the other hand, WLI used indirectly on impressions of the eroded dentin would overcome this limitation, and also the problem with collapse of the exposed organic matrix if the dentin dries out after erosion.

## Conclusion

With some adjustments like reference surface, the present findings support that the use of SRI in combination with WLI on impressions may be a promising strategy for clinical studies of erosive tooth wear. These instruments give accurate measurements of erosive tooth wear at initial and advanced stages respectively.

## Data Availability

The datasets generated or analysed during this study are available from the corresponding author on reasonable request.

## Abbreviations

ETW: Erosive Tooth Wear
OP: Opti Pen Handheld device
SH: Surface Hardness
SRI: Surface Reflection Intensity
TT: Table Top Device
WLI: White Light Interferometer
